# Diagnostic Value of a SARS-CoV-2 Rapid Test Kit for Detection of Neutralizing Antibodies as a Point-of-Care Surveillance Test

**DOI:** 10.1128/spectrum.00993-21

**Published:** 2022-03-07

**Authors:** Michele Mun Hei Chan, Ka-Yi Leung, Ricky Rui Qi Zhang, Danlei Liu, Yujing Fan, Matthew Ka Wa Khong, Anthony R. Tam, Honglin Chen, Kwok-Yung Yuen, Ivan F. N. Hung, Kwok-Hung Chan

**Affiliations:** a Department of Medicine, Li Ka Shing Faculty of Medicine, University of Hong Kong, Hong Kong Special Administrative Region, China; b Department of Microbiology, Li Ka Shing Faculty of Medicine, University of Hong Kong, Hong Kong Special Administrative Region, China; c Department of Medicine, Queen Mary Hospital, Hong Kong Special Administrative Region, China; d State Key Laboratory for Emerging Infectious Diseases, Li Ka Shing Faculty of Medicine, University of Hong Kong, Hong Kong Special Administrative Region, China; e Carol Yu Centre for Infection, Li Ka Shing Faculty of Medicine, University of Hong Kong, Hong Kong Special Administrative Region, China; University of Georgia

**Keywords:** antibody, convalescence, humoral response, immunization, rapid tests, SARS-CoV-2

## Abstract

Detection and tracking of antibodies play an increasingly prominent role in population surveillance and implementation of public health measures to combat the current coronavirus disease 2019 (COVID-19) pandemic, with much attention placed on developing commercial serological assays as point-of-care diagnostic tools. While many rapid diagnostic tests (RDTs) that detect severe acute respiratory syndrome coronavirus 2 (SARS-CoV-2) IgG and IgM antibodies have been evaluated, there is currently limited insight into detection of neutralizing antibodies (nAbs) by such modalities. Here, we evaluate performance characteristics of an RDT that detects SARS-CoV-2 IgG antibodies and, importantly, nAbs based on both infection- and vaccine-immunized cohorts by direct comparison to known antibody titers obtained from live virus microneutralization (VMN) assays. We further contextualize interpretations of band intensity of the RDT with reference to the World Health Organization (WHO) International Standard. We report a sensitivity of 94.37% and specificity of 92.50% for SARS-CoV-2 IgG detection and a sensitivity of 94.37% and specificity of 92.68% for nAbs. A limit of detection was determined as 3.125 IU/mL and 25.00 IU/mL, respectively, with reference to the WHO International Standard. We confirm that indication of nAb concentration, as elucidated by band intensity on the RDT, correlated with nAb titers defined by VMN assays and surrogate nAb assays. We additionally observe no cross-reactivity of the nAb test line to SARS-CoV-1 but report display of weak seropositivity for one sample on the SARS-CoV-2 IgG test line. Our study reveals promising performance characteristics of the assessed RDT, which implicates its usefulness in a wide range of diagnostic and epidemiological settings.

**IMPORTANCE** In the ongoing coronavirus disease 2019 (COVID-19) pandemic, antibody tests play an increasingly important role in detecting previous infection with severe acute respiratory syndrome coronavirus 2 (SARS-CoV-2) and monitoring of response to vaccinations. In particular, neutralizing antibodies have recently been demonstrated to be highly predictive of immune protection against symptomatic infection. Our study is the first to evaluate a rapid diagnostic test based on samples acquired from both recovered COVID-19 patients and individuals vaccinated for SARS-CoV-2, which detects neutralizing antibodies in addition to SARS-CoV-2 IgG. We report promising sensitivity, specificity, and cross-reactivity profiles, which implicate its usefulness in a wide range of settings as a diagnostic point-of-care tool to aid in curbing transmission and reducing mortality caused by COVID-19 symptoms.

## INTRODUCTION

Severe acute respiratory syndrome coronavirus 2 (SARS-CoV-2) continues to spread globally in pandemic proportions. With more than 175 million cases of infection documented since its emergence over 1 year ago, increasing evidence points to a protective role of postinfection immunity against reinfection and/or risk of severe disease outcomes (1). The advent of vaccines with promising efficacies ranging from 50% to 95% further adds a fascinating dimension to the immunological landscape against SARS-CoV-2 (2). While precise immune correlate(s) of protection against SARS-CoV-2 infection remain enigmatic, neutralizing antibodies (nAbs) have recently been demonstrated to be highly predictive of immune protection against symptomatic infection (3); thus, much attention has been placed on the development of antibody tests as a diagnostic tool to curb transmission and reduce mortality caused by coronavirus disease 2019 (COVID-19) symptoms.

Unlike nucleic acid amplification tests (NAATs), which only inform recent infections, antibody tests additionally allow for diagnosis of past infections and reveal vaccine-induced humoral immune responses (4). This highlights its epidemiological significance as a point-of-care test (POCT) in surveillance and implementation of public health measures by differentiating between individuals who are protected against or vulnerable to SARS-CoV-2 infection.

Various in-house and commercial antibody tests have been developed based on recombinant SARS-CoV-2 spike (S), S1 subunit, receptor-binding domain (RBD), and nucleocapsid (N) antigens to detect IgG antibodies (5). However, developing modalities to test for neutralizing antibodies is far more challenging. The current gold standard approach is the use of neutralization assays with replication-competent SARS-CoV-2, which is limited by speed and safety due to its requirement of biosafety level 3 facilities. Our study evaluates a rapid diagnostic test (RDT) that detects both anti-RBD IgG antibodies and neutralizing antibodies that block the interaction between RBD and human angiotensin converting enzyme 2 (ACE2).

The potential translational value of an antibody POCT is heavily dependent on its sensitivity and specificity. While many studies have emerged to validate performance of antibody POCTs based on postinfection antibody responses (5–7), the sensitivity of this particular test kit was determined by the manufacturer using samples immunized by vaccination, introducing potential points of discrepancies when attempting to evaluate their translational value as POCTs. Our current study therefore aims to contextualize this POCT in both the postinfection and postvaccination setting. Our work additionally evaluates the potential of its cross-reactivity with convalescence to SARS-CoV-1.

## RESULTS

### Overview of test kit interpretation and limit of detection.

Our selected RDT detects both anti-RBD IgG antibodies and nAbs using immunocapture-liquid chromatography (see Materials and Methods), with the color intensity of each band reflecting the concentration of the respective antibody type.

**FIG 1:**
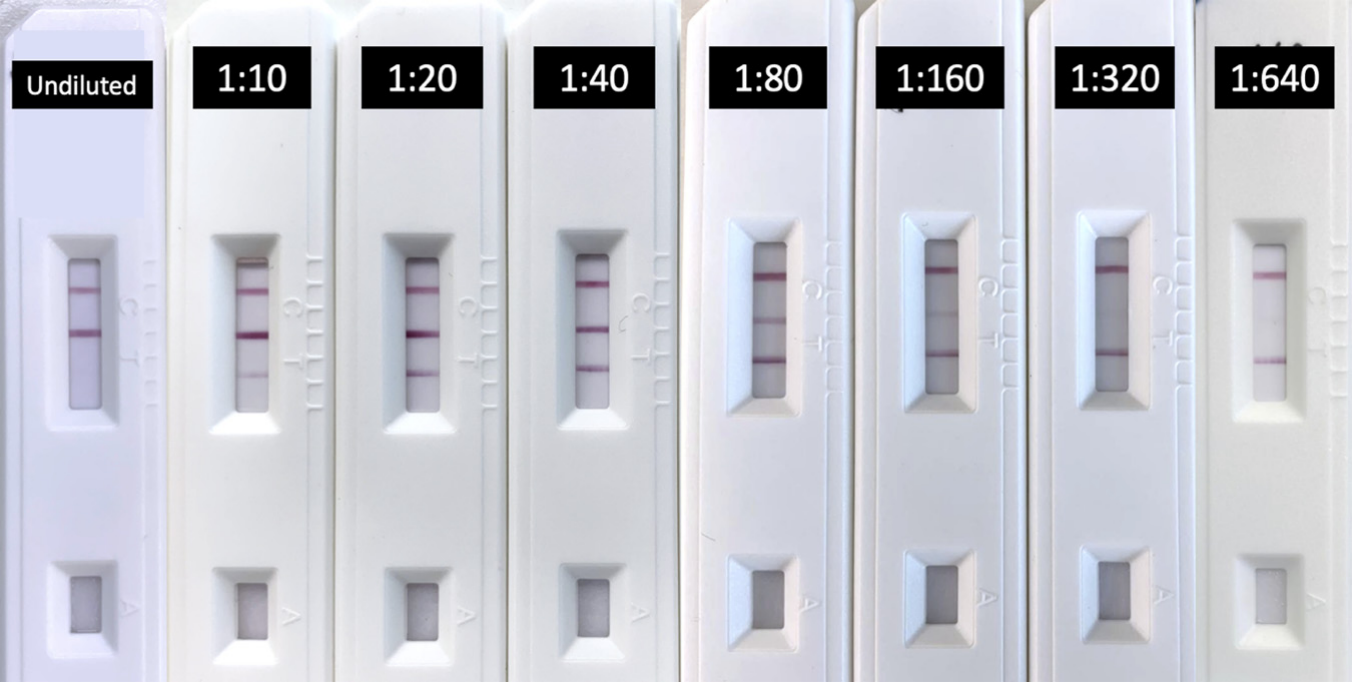
Test kit interpretation with reference to WHO International Standard units. The cassette labeled “undiluted” was tested with unmanipulated International Standard SARS-CoV-2 human immunoglobulin sample as provided by the WHO. From left to right, a sample was subjected to a 10-fold dilution (1:10) and subsequently to 2-fold dilutions until a dilution factor of 1:640 was reached.

We first set out to contextualize interpretation of band intensity in terms of the International Standard for human anti-SARS-CoV-2 immunoglobulin. To this end, we evaluated the test kit using the International Standard sample provided by the World Health Organization (WHO) in 2-fold dilutions (Table S2 in the supplemental material). Accordingly, we observed a gradual decrease in band intensity on the test line for SARS-CoV-2 IgG and an incremental increase in band intensity for nAbs. Of note, changes in the latter were less clear from dilutions of 1:40 and beyond (Fig. 1), with the color intensity of the nAb band largely comparable to the control band; thus, 25.00 IU/mL was determined as the limit of detection for nAbs.

We next sought to establish a standardized approach to interpreting band intensity. A four-point scale was used to denote the test result for both bands: negative, weak positive (weak+), moderate positive (moderate+), and strong positive (strong+). For SARS-CoV-2 IgG, band intensities comparable to those of 1:160, 1:80, and 1:40 were defined as weak+, moderate+, and strong+, respectively. For nAbs, intensities comparable to those of 1:40, 1:20, and 1:10 were similarly defined as weak+, moderate+, and strong+ and used as reference points for all subsequent analysis.

At a dilution of 1:320, the band for SARS-CoV-2 was barely visible; thus, 3.125 IU/mL was determined as the limit of detection for SARS-CoV-2 IgG.

### Performance characteristics of RDT.

**FIG 2:**
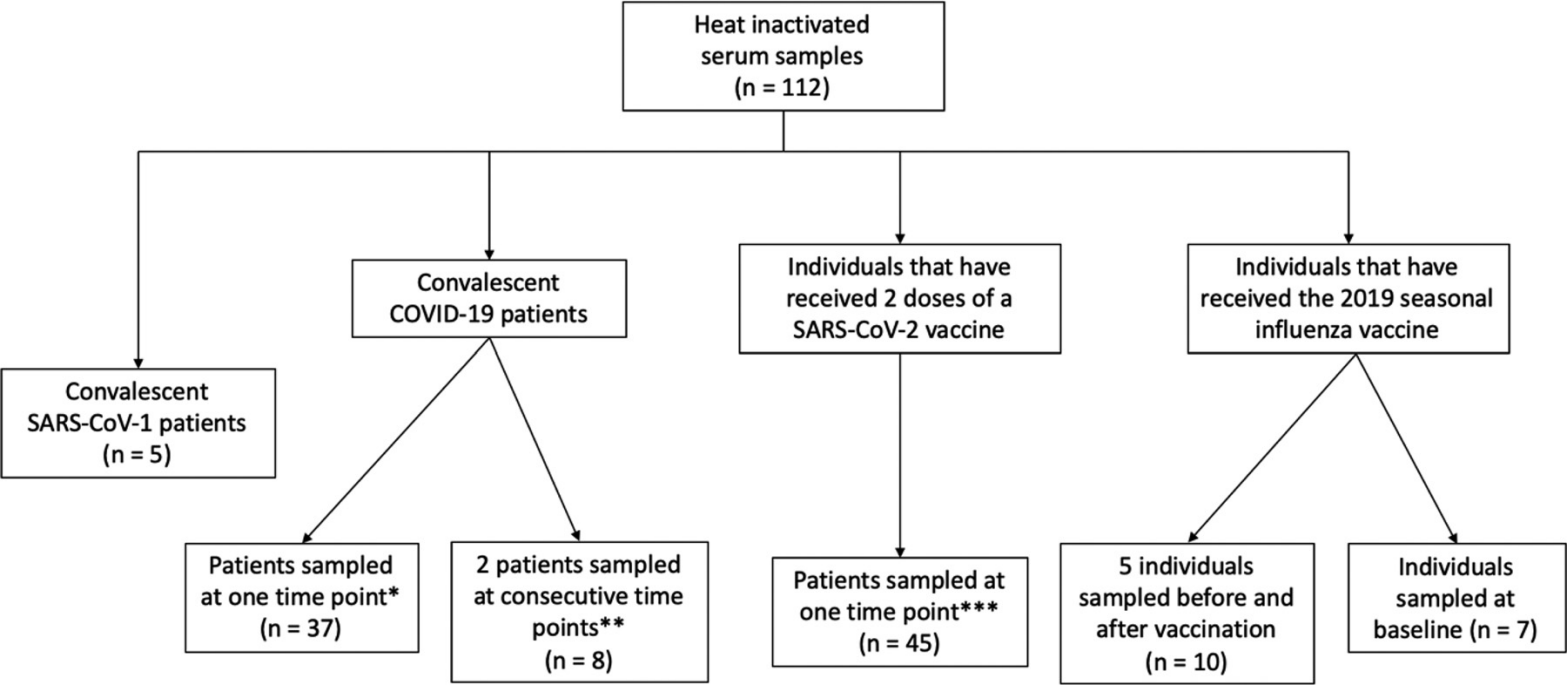
Schematic overview of samples. The one asterisk (*) indicates that samples were collected from 37 convalescent COVID-19 patients at least 30 days after recovery. Recovery was defined as the day patients complete two consecutive negative PCR tests. Two asterisks (**) indicate that 5 samples were collected from one convalescent COVID-19 patient over 15 days. Three samples were collected from one convalescent COVID-19 patient over 7 days. Three asterisks (***) indicate that samples were collected from 45 vaccinated individuals at least 21 days, and up to 56 days, after they have received their second dose.

One hundred and twelve heat-inactivated serum samples were collected to determine sensitivity and specificity of the RDT (Fig. 2).

Importantly, evaluation using SARS-CoV-2 IgG test line conferred differing sensitivities and specificities to nAb test line. Detection of SARS-CoV-2 IgG was 94.37% sensitive (95% confidence interval [95% CI] of 86.20 to 98.44) and 92.50% specific (95% CI of 79.61 to 98.43) (Table 1), with 3 false-positive and 4 false-negative results. In comparison, the test kit had an equal sensitivity of 94.37% (95% CI of 86.20 to 98.44) and marginally higher specificity of 92.68% (95% CI of 80.08 to 98.46) for detection of nAbs (Table 2), with 3 false-positive and 4 false-negative results.

**TABLE 1:** Performance characteristics of RDT SARS-CoV-2 IgG test line based on nAb results determined by VMN assay (*n* = 112)

Performance characteristics	Value	95% CI
Sensitivity	94.37%	86.20% to 98.44%
Specificity	92.50%	79.61% to 98.43%
Positive likelihood ratio	12.58	4.23 to 37.42
Negative likelihood ratio	0.06	0.02 to 0.16
Positive predictive value	95.71%	88.25% to 98.52%
Negative predictive value	90.24%	78.05% to 96.01%

**TABLE 2:** Performance characteristics of RDT nAb test line based on nAb results determined by VMN assay (*n* = 112)

Performance characteristics	Value	95% CI
Sensitivity	94.37%	86.20% to 98.44%
Specificity	92.68%	80.08% to 98.46%
Positive likelihood ratio	12.9	4.33 to 38.39
Negative likelihood ratio	0.06	0.02 to 0.16
Positive predictive value	95.71%	88.24% to 98.52%
Negative predictive value	90.48%	78.51% to 96.11%

Antibody POCT evaluations in the current literature are largely based on samples collected from COVID-19 patients (5–7), while our study includes individuals immunized by vaccination. We therefore performed subgroup analysis to elucidate potential differences, if any, between performance characteristics of the RDT based on convalescent or postvaccination serum samples. We report a sensitivity of 100% and specificity of 90.62% for convalescent COVID-19 patients, with 3 false-positive results. Postvaccination samples had a lower sensitivity of 88.89% but higher specificity of 100.00%, with 4 false-negative results (Table 3).

**TABLE 3:** Subgroup analysis of performance characteristics of RDT nAb test line based on nAb results determined by VMN assay

Performance characteristics	Convalescent COVID-19 patients (*n* = 45)		Vaccinated individuals (*n* = 45)	
	Value	95% CI	Value	95% CI
Sensitivity	100.00%	90.00% to 100.00%	88.89%	73.94% to 96.89%
Specificity	90.62%	74.98% to 98.02%	100.00%	89.11% to 100.00%
Positive likelihood ratio	10.67	3.63 to 31.32	–	–
Negative likelihood ratio	0	–^*a*^	0.11	0.04 to 0.28
Positive predictive value	92.11%	79.89% to 97.16%	100.00%	–
Negative predictive value	100.00%	–	88.89%	76.05% to 95.27%

a –, not applicable.

We subsequently compared the performance of the assessed RDT with that of the surrogate nAb assay (YHLO Biotech Co., Ltd.). Interestingly, the surrogate nAb assay had a higher sensitivity of 100% (95% CI of 94.79 to 100.00) but a lower specificity of 80.95% (95% CI of 58.09 to 94.55) (Table S3), reflective of 4 false-positive results.

### Indication of nAb concentration by RDT correlates with titers determined by laboratory immunoassays.

Manufacturer’s instructions of this test kit state that a lighter band on the nAb test line indicates higher concentrations of neutralizing antibodies. We therefore sought to confirm whether nAb concentration, as indicated by band intensity, correlated with nAb titers determined by well-validated immunoassays in the laboratory.

**FIG 3:**
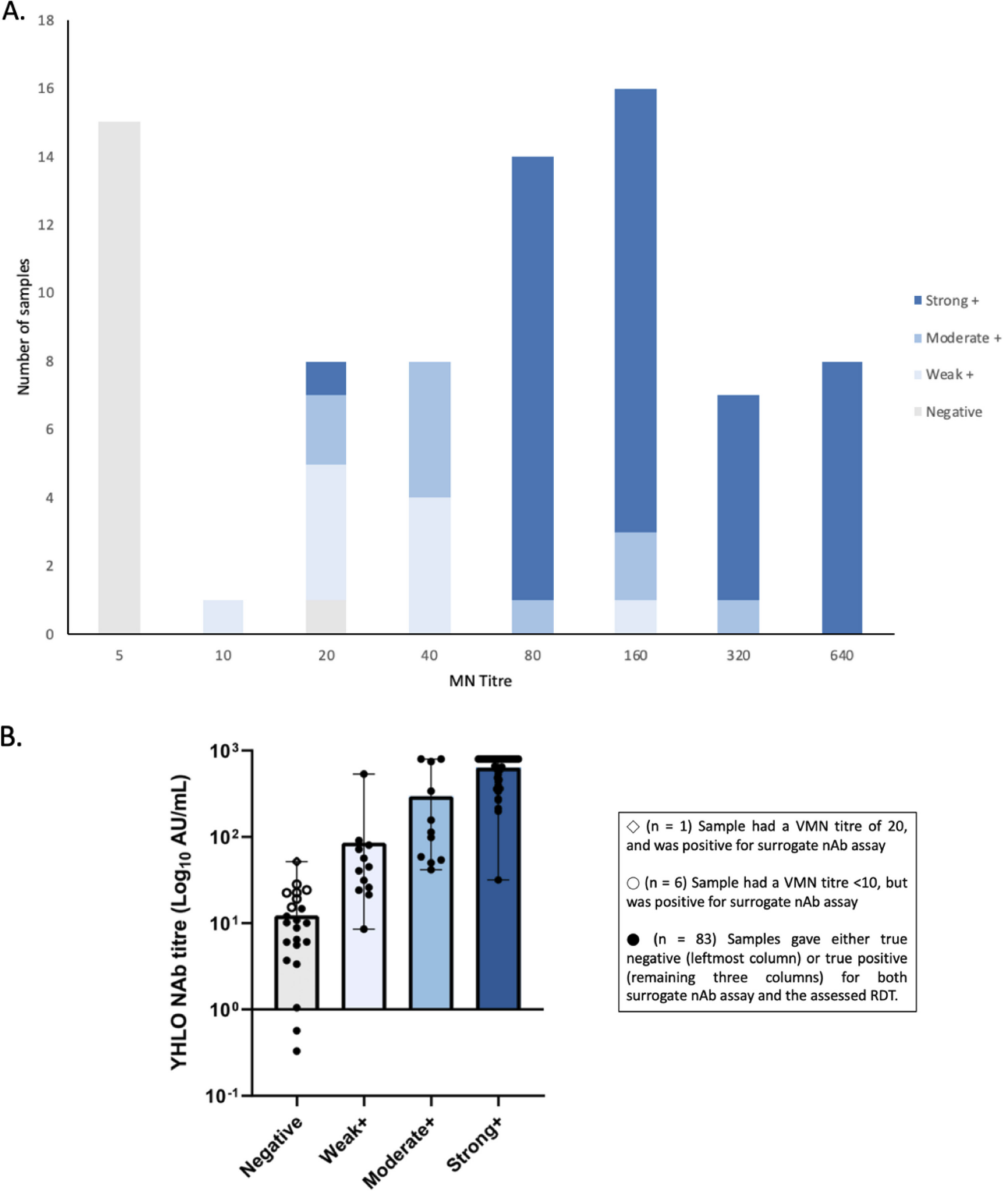
Performance evaluation by comparison of nAb test line results with defined nAb results (*n* = 90). Results of the nAb test line were determined by the following four-point scale: “negative”, “weak+”, “moderate+,” and “strong+” based on band intensity. (A) Comparison of nAb test line results with nAb titers ranging from <10 to >320, as determined by an in-house MN assay. (B) Comparison of nAb test line results with nAb titers as determined by an in-house CLIA by YHLO iFlash 1800; titer values are displayed on a log_10_ scale. Each individual data point represents one sample. The graph shows individual data points, mean, and range.

For each sample, we compared interpretation of the nAb test line to nAb titers as determined by virus microneutralization (VMN) assays (Fig. 3A). In keeping with the specificity of the nAb test line of 100%, we report that all 15 samples that had MN titers below 10 were also interpreted as “negative” by the RDT. Of note, samples with MN titers of 20 and 40 displayed various band intensities. Samples with MN titers of 80 and above were mostly interpreted as “strong+” by the RDT.

We subsequently compared the same data set acquired from interpretation of the nAb test line to nAb titers quantified by the surrogate nAb assay (YHLO Biotech Co., Ltd.). We report that the mean nAb titer of samples interpreted as “strong+” was the highest, followed by a lower mean nAb titer in the “moderate+” samples. The mean nAb titer of samples interpreted as “weak+” was further decreased (Fig. 3B). Notably, 7 samples interpreted by the nAb test line as “negative” displayed nAb titers higher than the seropositivity cutoff of 15 arbitrary units (AU)/mL.

Collectively, these results suggest that indication of nAb concentration by the assessed RDT, as interpreted from band intensity of the test line, correlates with nAb titers determined by VMN assay and the surrogate nAb assays.

### Calibration of RDT interpretation with defined nAb titer values.

We next aimed to calibrate the qualitative RDT interpretations with quantitative nAb titer values, as determined by VMN assay and the surrogate nAb assay. According to evaluation using the WHO International Standard (Fig. 1 and Table 4), negative RDT results based on the nAb test line had concentrations of 25.00 IU/mL or below. An RDT interpretation of “weak+” and “moderate+” fell between ranges of 25.00 to 49.99 IU/mL and 50.00 to 99.99 IU/mL, respectively. An RDT interpretation of “strong+” had concentrations of 100.00 IU/mL or above.

**TABLE 4:** Calibration of qualitative RDT interpretation calibration based on WHO International Standard sample in comparison to mean nAb titer determined by surrogate nAb assay

RDT interpretation (nAb test line)	Calibration by WHO International Standard (IU/mL)	Mean nAb titer by surrogate nAb assay (IU/mL)
Negative	25.00 or below	29.59
Weak+^*a*^	25.00–49.99	206.10
Moderate+	50.00–99.99	711.44
Strong+	100.00 or above	1,525.4

a +, positive.

However, we report that mean nAb titer values, as measured by the surrogate nAb assay, did not fall in the previously established range. While the mean neutralizing antibodies (nAb) titers for “negative” (29.59 IU/mL) were comparable to the International Standard, those of “weak+” and “moderate+” were significantly higher than the calibrated range.

### RDT does not display cross-reactivity to anti-SARS-CoV-1.

Antibodies against SARS-CoV-1 structural proteins have been shown to display cross-reactivity with SARS-CoV-2, as demonstrated by microscopy and immunoblotting (8). However, recent studies suggest that cross-neutralization of live, patient-derived strains of SARS-CoV-1 and SARS-CoV-2 are rare in contrast (9).

We therefore aimed to determine whether the nAb test line of the RDT was cross-reactive to anti-SARS-CoV-1 antibodies. To this end, we tested convalescent SARS-CoV-1 samples and observed no difference in band intensity between the nAb test line and control line, indicating that anti-SARS-CoV-1 antibodies were undetectable by the assessed RDT. Interestingly, the SARS-CoV-2 IgG test line showed weak seropositivity for one of the samples.

### Subjectivity with interpretation.

To test for consistency among different cassettes, we used samples from individuals that were recruited in a trial for the 2019 seasonal influenza vaccine as negative controls. While all cassettes could be clearly interpreted as “negative” on the SARS-CoV-2 IgG test line, we observed slight inconsistencies in band color and intensity of the nAb test line compared to the control line (Fig. S1). This may be significant in samples containing smaller concentrations of nAb, where interpretation of the nAb test line as “negative” or “weak+” becomes subjective to individual judgement.

## DISCUSSION

Antibody tests play an increasingly important role in detecting previous infection to SARS-CoV-2 and monitoring of response to vaccinations. While many commercially available RDTs can detect IgG and IgM antibodies (5, 10), our study evaluates a test kit that can detect nAbs in addition to SARS-CoV-2 IgG antibodies. We report that the assessed RDT had a sensitivity of 94.37% and specificity of 92.50% for detection of SARS-CoV-2 IgG and a sensitivity of 94.37% and specificity of 92.68% for detection of nAbs, with the limit of detection determined as 3.125 IU/mL and 25.00 IU/mL, respectively. We confirm that indication of nAb concentration, determined by band intensity of the RDT, correlated with nAb titers elucidated by in-house VMN and surrogate nAb assays.

To place the performance of this RDT in the context of other currently available surrogate immunoassays, we assessed results from an automated one-step competitive chemiluminescence assay (CLIA) in parallel (YHLO, iFlash 1800). The iFlash 1800 analyzer was recently used in a study of convalescent COVID-19 patients in Italy for the detection of anti-SARS-CoV-2 IgM and IgG antibodies, where specificity was determined at 100% for cutoff values of 7.1 AU/mL and above. Contrastingly, we report a specificity of 80.95% at a cutoff of 15 AU/mL with 4 false-positive results, which was also notably lower than that of the RDT (11). We acknowledge that a direct comparison cannot be made; the Italian study assessed a CLIA detecting IgM and IgG, while our study investigated a CLIA testing for nAbs. However, we reason that our cohort of vaccinated individuals, not present in their study, may have also contributed to differences in specificity. This suggests a need for future refinement of the seropositivity threshold to better cater for immunity acquired through natural infection and vaccination. Indeed, our subgroup analysis revealed differing sensitivities and specificities between convalescent and vaccinated cohorts.

In addition, we note slight discrepancies between our evaluated performance characteristics based on vaccinated patients compared to values provided on the manufacturer’s website, which stated a notably higher sensitivity of 92.47% and minimally lower specificity of 99.68%. All participants in the manufacturer’s evaluation received the BioNTech mRNA vaccine, while a proportion of individuals in our study were vaccinated with inactivated virus modalities (Sinovac and BIBP-CorV). Given recent evidence demonstrating significant differences in immunogenicity between mRNA and inactivated vaccines (12), we speculate that variation in vaccination type may have contributed to the differing sensitivities. However, we also reason that sample size may have been a contributing factor, as the manufacturer reported seven false negatives, which was, interestingly, three more than our study.

Further, calibration of the RDT nAb test line interpretation with the International Standard revealed a notable mismatch with mean titers determined by the surrogate nAb assay. We recognize that our calibration with the International Standard only provides an approximate guide, which could also contribute to the observed mismatch with mean titers from the surrogate nAb assay.

Neutralizing antibodies are known to be a major correlate of protective immunity to SARS-CoV-2 infection, targeting both RBD and non-RBD epitopes to block viral entry through multiple mechanisms (13, 14). A major advantage of the RDT assessed in our study is its ability to detect nAbs with reasonable sensitivity and high specificity. However, we note slight inconsistencies in band color and intensity of the nAb test line among different cassettes, thus interpretation may vary due to individual subjectivity, especially for lower nAb concentrations. Furthermore, the limit of detection was determined to be 25.00 IU/mL for the RDT, which is higher than that of our in-house VMN assay of 6.250 IU/mL (unpublished data). Therefore, we reason that while this RDT cannot replace microneutralization assays in the accurate determination of nAb titer, the RDT provides a quicker and safer tool that can be used in a wider range of settings as a POCT.

We observe no cross-reactivity to SARS-CoV-1 in terms of nAbs but report that the SARS-CoV-2 IgG test line showed weak seropositivity for one sample. We postulate that the weak IgG band observed was due to detection of anti-NP antibodies present in the convalescent SARS-CoV-1 sample, which have been demonstrated to display substantial cross-reactivity against SARS-CoV-2 in the literature (8). In the context of preexisting immunological memory to the previously pandemic strain of SARS-CoV-1, assessment of cross-reactivity is particularly important. Future studies should additionally investigate potential cross-reactivity with other pandemic coronaviruses, such as Middle East respiratory syndrome coronavirus (MERS-CoV) and seasonal coronaviruses OC43, 229E, HKU1, and NL63, which will provide further insight into the suitability of this RDT as a POCT in various settings.

Evaluations of RDTs in the current published literature have mainly used convalescent patient samples. Our study additionally includes validation from vaccinated individuals. With an increasing proportion of individuals vaccinated against SARS-CoV-2, we propose this RDT as an appropriate tool to distinguish between immunologically naive individuals and those that are immunized through natural infection, vaccination, or both. Potential uses include deployment at travel borders and quarantine sites to drive evidence-based implementation of public health measures and large-scale tracking of infection- and vaccination-acquired immunity, which can be used to inform future vaccination strategies.

In particular, rapid diagnostic assays have recently been proposed as useful tools to be administered at border crossings (15), albeit with a focus on detection of SARS-CoV-2 antigen. We additionally put forward our assessed RDT as a potentially suitable candidate for use at border control in airports. A positive nAb result from the RDT may have distinct implications for different countries. These travelers may enjoy shortening or exemption of a quarantine order following arrival or be given the option of quarantining at home instead of at designated sites. However, we acknowledge the possibility of false positives given the RDT’s specificity of 92.68% in detection of nAbs. We therefore recommend a positive RDT result to be treated conservatively and with caution. For example, countries may find it appropriate for travelers with a positive nAb to be subjected to further confirmation by laboratory-based serological assays before full quarantine exemption.

These limitations notwithstanding, we believe that this RDT offers a more affordable modality of serological testing that can be performed without the need for sophisticated equipment or specialized laboratory staff. The element of efficiency is another attractive advantage. As a point-of-care test outside the laboratory, the manufacturer recommends collection of finger prick capillary blood from individuals to complete the test. We further report a completion time of 30 min using existing samples. Taken together, we estimate no more than 60 min from sample collection to reporting of results.

However, while the RDT assessed in our study appears to hold promising translational value, it also bears intrinsically limiting factors. One significant drawback is the aforementioned element of subjectivity in the interpretation of the nAb test line, rendering results difficult to standardize between different observers, particularly for low nAb titers. Another major caveat is the emergence of variants of concern (VOCs), which are becoming the dominant transmitting strains in many countries (16). There is now robust evidence to support the changing antigenicity of VOCs, which may contribute to immune escape to a degree that can jeopardize infection- or vaccine-acquired immune protection (17).

In conclusion, our study is the first to evaluate an RDT based on both postinfection and postvaccination serum samples, which detects nAbs in addition to SARS-CoV-2 IgG. We report promising sensitivity, specificity, and cross-reactivity profiles, which implicate its usefulness in a wide range of settings. Further studies into the level and duration of infection- and vaccination-acquired immunity will provide more conclusive immune correlates of protection, which will add epidemiolocal insight into the implications of a positive result on this RDT, especially in the context of emerging VOCs.

## MATERIALS AND METHODS

### Samples.

A total of 112 serum samples were used to evaluate the performance of the rapid antibody POCT (JOYSBIO, Tianjin, China) by direct comparison to live virus microneutralization (VMN) and surrogate neutralization assay data.

Forty-five samples were collected from 39 convalescent COVID-19 patients from July to November 2020, with consecutive samples from 2 recovered patients. Forty-five samples from 45 individuals with no known previous SARS-CoV-2 infections were collected at least 14 days, and up to 56 days, after receiving the second dose of a SARS-CoV-2 vaccine (Table S1 in the supplemental material) from March to April 2021. To test for cross-reactivity, 17 samples from 12 individuals collected at baseline and 21 days after receiving the 2019 seasonal influenza vaccine and 5 samples from convalescent SARS-CoV-1 patients were used. All samples were heat inactivated by incubation at 56°C for 30 min before use.

### Antibody rapid test.

The SARS-CoV-2 IgG/neutralizing antibody rapid test kit (Colloidal Gold; JOYSBIO, Tianjin, China) was evaluated. All testing was carried out in a single determination according to the manufacturer’s instructions.

Ten microliters of serum was needed to fill the square hole of the test cassette using a calibrated pipette. Three precoated lines mark the control line (C) and the test lines for SARS-CoV-2 IgG (1) and neutralizing antibodies (nAbs) (2). Colloidal gold-labeled chicken IgY antibody bound to goat anti-chicken IgY antibody coated on the C line acts as the positive control. Mouse anti-human IgG antibody coated on test line 1 captures human IgG anti-nucleoprotein (NP) and anti-spike (S) protein antibodies in the sample to form a colored band. Colloidal gold-labeled recombinant SARS-CoV-2 RBD and human ACE2 coated on line 2 detects the presence of nAbs. Any unbound recombinant RBD as well as RBD bound to nonneutralizing antibody is captured on test line 2, which forms a colored band. The band appears lighter with increasing concentration of nAbs. A band that is darker or equal to the C line is interpreted as a negative result.

Seventy microliters of diluent was needed to fill the circular hole of the test cassette, using either a calibrated pipette or the prefilled single-use dropper provided. The test cassette should be read 25 to 30 min after addition of sample and diluent.

### Live virus microneutralization assay.

SARS-CoV-2 virus culture and MN were performed in a biosafety level 3 facility, as previously described (18). In brief, 2-fold serial dilutions of serum samples were prepared with minimum essential medium (MEM) with 1% fetal bovine serum (FBS) in 50 μL and mixed with replication-competent SARS-CoV-2 in 50 μL to give a final dilution of 1:10 in 100 μL and a virus titer of 100 50% tissue culture infective dose (TCID_50_). This serum-virus mixture was incubated for 1.5 h at 37°C before it was added to Vero E6 cells incubated at 37°C in a 5% CO_2_ incubator. Virus-induced cytopathic effect was examined under inversion microscopy after 4 to 5 days of incubation. MN antibody titer was determined by the highest dilution with 50% inhibition of cytopathic effect. An MN titer of ≥10 was considered positive. For statistical analysis, titers of <10 were treated as 5; titers of >320 were treated as 640.

### Chemiluminescent microparticle immunoassay.

Testing of nAbs against SARS-CoV-2 RBD was performed using the iFlash-2019-nCoV NAb assay (Shenzhen YHLO Biotech Co. Ltd., China), and a one-step competitive CLIA was performed on the YHLO iFlash 1800 chemiluminescence immunoassay (YHLO Biotech Co., Ltd., China). The iFlash-2019-nCoV nAb assay was performed according to manufacturer’s instructions (YHLO Biotech Co., Ltd.). Briefly, serum samples were put on the sample rack and placed in the sample loading area. The test-specific parameters stored in the barcode on the reagent pack are read in (2019-nCoV RBD antigen-coated paramagnetic microparticles, acridinium ester-labeled ACE2 conjugate, sample-treating agent). The iFlash System performs all the functions automatically and calculates the results when the run is initiated. The signal from the chemiluminescent reaction is measured as relative light units (RLUs), which exist in an inverse relationship with the amount of 2019-nCoV nAbs present in the sample. Results are then determined via a calibration curve. The cutoff for seropositivity is 15 AU/mL. The maximum measurable result is 800 AU/mL (1 AU = 2.4 IU).

### Statistical analysis.

Sensitivities, specificities, and positive and negative predictive values with Wilson score 95% confidence intervals were calculated using 2021 MedCalc Software, Ltd. Graphs were made using 2019 Microsoft Excel (Fig. 3A) and GraphPad Prism version 9 (Fig. 3B).

### Ethical statement.

The present study was approved by the Institutional Review Board of the University of Hong Kong/Hospital Authority Hong Kong West Cluster (UW13-372).
