# Supplementary material for: Diagnostic Value of a SARS-CoV-2 Rapid Test Kit for Detection of Neutralizing Antibodies as a Point-of-Care Surveillance Test
Source: Microbiol Spectr. 2022 Mar 7;10(2):e00993-21. doi: 10.1128/spectrum.00993-21 (PMC9045162; doi:10.1128/spectrum.00993-21)
Supplement: SUPPLEMENTAL FILE 1 — Supplemental material. Download SPECTRUM00993-21_Supp_1_seq8.pdf, PDF file, 0.5 MB [file spectrum00993-21_supp_1_seq8.pdf]

Supplementary Data

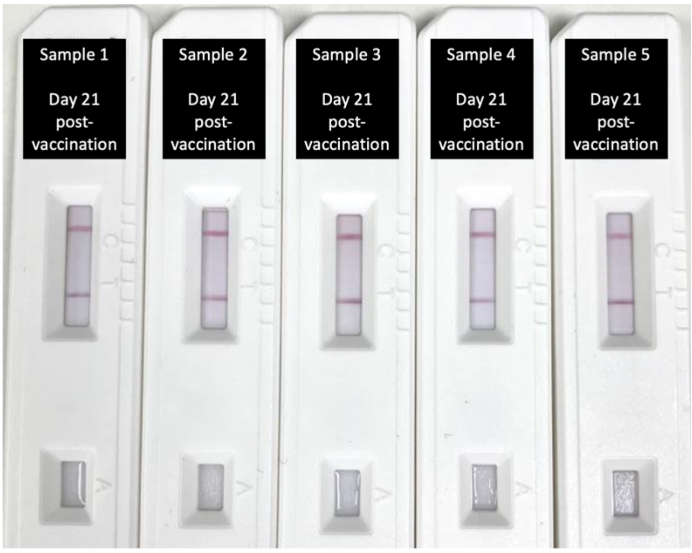

Supplementary Figure 1. Five representative cassettes tested with serum samples from five individuals 21 days after receiving their 2019 seasonal influenza vaccination. Samples were collected before the start of the COVID-19 pandemic.

Vaccine	Number of vaccinees
COMINARTY® BNT162b2 (BioNTech/ Fosun Pharma)	33
CoronaVac (Sinovac Biotech)	11
BIBP-CorV (Sinopharm)	1

Supplementary Table 1. SARS-CoV-2 vaccines received by the vaccinated cohort (n=45).

Dilution ratio	International Units per mL (IU/mL)
Undiluted	1000
1:10	100.0
1:20	50.00
1:40	25.00
1:80	12.50
1:160	6.250
1:320	3.125

Supplementary Table 2. Reference table showing dilution ratio of the International Standard SARS-CoV-2 human immunoglobulin sample with its corresponding antibody titre in IU/mL.

	Value	95% CI
**Sensitivity**	100.00%	94.79% to 100.00%
**Specificity**	80.95%	58.09% to 94.55%
**Positive Likelihood Ratio**	5.25	2.17 to 12.86
**Negative Likelihood Ratio**	-	-
**Positive Predictive Value**	94.52%	87.72% to 97.66%
**Negative Predictive Value**	100.00%	

Supplementary Table 3. Performance characteristics of surrogate nAb assay based on nAb results determined by VMN. (n = 96)
